# Effects of Dietary Phosphatidylcholine Supplementation on Growth Performance, Antioxidant Capacity, Fatty Acid Composition, and Lipid Metabolism of Juvenile *Eriocheir sinensis*-Fed Different Oil Sources

**DOI:** 10.1155/anu/5627355

**Published:** 2025-02-12

**Authors:** Zhideng Lin, Xiaodan Wang, Xianyong Bu, Qincheng Huang, Han Wang, Erchao Li, Jianguang Qin, Liqiao Chen

**Affiliations:** ^1^Laboratory of Aquaculture Nutrition and Environmental Health, College of Life Sciences, East China Normal University, 500 Dongchuan Road, Shanghai 200241, China; ^2^College of Marine Science, Ningde Normal University, Fujian 352100, China; ^3^Xianghu Laboratory, Hangzhou 311231, China; ^4^College of Science and Engineering, Flinders University, Adelaide 5001, South Australia, Australia

**Keywords:** *Eriocheir sinensis*, fatty acids, lipid metabolism, oil sources, phosphatidylcholine

## Abstract

The present study investigates the effects of dietary phosphatidylcholine (PC) deficiency and its addition on growth and physiological and biochemical indicators of juvenile *Eriocheir sinensis* under different oil sources. There were no significant differences in the growth and feed utilization between the vegetable oils and fish oil (FO) groups under PC-devoid conditions. In contrast, the FO and perilla oil (PO) groups showed better growth-promoting effects and higher feed utilization than the safflower oil (SO) and olive oil (OO) groups under 3% PC-added condition. Both dietary PC and oil sources (FO or PO) could inhibit lipid accumulation of the whole crab, and dietary PC also observably facilitated whole-body protein deposition. In addition, dietary FO and PO increased the burden of the antioxidant system and the risk of lipid peroxidation in juvenile *E. sinensis*. Meanwhile, diets supplemented with PC effectively alleviated oxidative stress and lipid peroxidation caused by dietary FO and PO. The composition of fatty acids in muscle and hepatopancreas was positively associated with that in diets. Compared with SO and OO, FO and PO significantly reduced the lipid deposition in the hepatopancreas at 3% PC supplementation, possibly because FO and PO formed new physiological-active PC contained n-3 polyunsaturated fatty acids (PUFAs) with dietary PC through activating PC remodeling reaction, and promoting fatty acid utilization, and finally inhibiting the lipid accumulation in the hepatopancreas. This study indicates that FO and PO are better lipid sources (LSs) for *E. sinensis*, providing alternative oil sources in the crab diet in combination with PC supplementation.

## 1. Introduction

Lipids include neutral lipids (mainly triglycerides and cholesterol) and polar lipids (mainly phospholipids), which are essential nutrients for various aquatic animals [1–3]. Emerging studies have indicated that the source, composition, and concentration of lipids in diets can affect the growth, immunity, and development of aquatic animals and the nutritional composition and meat quality of aquatic products [3–9]. Besides, suitable lipid nutrition can improve dietary protein utilization, reduce feed costs, and increase feeding efficiency [6]. In addition to cholesterol, neutral lipids primarily exist in animal and vegetable oils. Fish oil (FO) contains abundant highly unsaturated fatty acids (HUFAs) and is the most common animal oil in aquatic diets. However, because of the depletion of fishery resources, FO production has been unable to meet the rapid development of the aquaculture industry. Therefore, many studies have been performed to find FO alternatives like vegetable oils [10]. Compared with FO, vegetable oils are widely available and inexpensive, which may be a viable alternative. The essential distinction between vegetable oil and FO is the difference in composition of fatty acids, and vegetable oils are usually deficient in physiological-active HUFA. Recently, many studies have been performed to evaluate the effects of FO replacement with vegetable oil, and some found that the substitution of vegetable oils for FO did not affect growth performance, feed utilization, or health status [11, 12]. However, most teleost studies showed that excessive dietary vegetable oil often causes abnormal lipid accumulation, growth inhibition, and reduced immunity [13–15]. Therefore, it is imperative to seek effective nutritional means to reduce the adverse impacts of replacing FO with vegetable oils.

The addition of dietary phospholipids can satisfy the demand for polar lipids for aquatic animals, especially for fast-growing larvae and juveniles [16]. Based on the structures of polar heads, phospholipids are mainly divided into phosphatidylserine (PS), phosphatidylethanolamine (PE), phosphatidylcholine (PC), and phosphatidylinositol (PI) [1]. Phospholipids have positive effects on development [16, 17], anti-inflammation [18], and energy metabolism [4, 5] for aquatic animals. Currently, the available phospholipids are primarily obtained from plants (soybean phospholipids, sunflower phospholipids, and rapeseed/canola phospholipids), land animals (egg yolk phospholipids), and marine animals (krill phospholipids and fishmeal) [1, 16, 19]. In addition to the difference in composition, the most significant discrepancy among these phospholipid sources is the type of fatty acids bound to the molecular structure of phospholipids. Compared with phospholipids extracted from plants and land animals, marine phospholipids connect more n-3 polyunsaturated fatty acids (PUFAs) on the sn-2 sites. Phospholipids binding with different fatty acids can affect their bioactive function, especially sn-2 sites connected with different PUFAs [20]. For example, marine phospholipids rich in n-3 PUFA possess more growth-promoting and lipid-lowering effects than those from plants and land animals (lack n-3 PUFA) [4, 16, 21–24]. In addition, dietary fatty acids can change the fatty acid composition on the sn-2 sites of phospholipids [25], and dietary phospholipids can promote HUFA accumulation in tissues [26–28], suggesting that a close metabolism relationship may exist between fatty acids and phospholipids. Currently, studies have more focused on investigating interactional relationships between phospholipids and cholesterol [29, 30], choline [31], or lipid levels [32, 33], while few have reported the relationship between phospholipids and fatty acids in different oil sources [34, 35].

As a crustacean with high economic value, the Chinese mitten crab (*Eriocheir sinensis*) has been widely cultured in China. Due to improved breeding and feeding technology, *E. sinensis* has become the most productive crab species in China, with an annual yield of 815,318 tons in 2022 [36]. In the past, we have determined the effects of different phospholipid sources [4, 5], the interaction of phospholipids and lipid levels [33], as well as different PC levels [28] on the growth, gonad development and lipid metabolism of *E. sinensis*. We have reported that marine phospholipids rich in n-3 HUFA show more powerful functions in promoting growth, gonad development, and lipid utilization, and dietary PC can effectively facilitate HUFA accumulation in tissues, indicating that fatty acids and phospholipids exist in a close relationship. Therefore, the present study aims to investigate further the interactive effects of different oil sources and PC (a representative of phospholipids) on growth and physiological and biochemical indicators of *E. sinensis*. These results may contribute to selecting appropriate lipid sources (LSs) from a phospholipid perspective for *E. sinensis* and increasing the knowledge of lipid nutrition in crustaceans.

## 2. Materials and Methods

### 2.1. Experimental Diets

Four oil sources and two PC levels were used to design a 2 × 4 factorial experiment for producing eight experimental diets with 35% crude protein and 9% total lipid. The two PC levels were devoid and 3%, respectively [28]. The four oil sources were olive oil (OO), perilla oil (PO), FO, and safflower oil (SO), respectively, and each oil source possessed a typical composition of fatty acids. OO was rich in oleic acid (OA, C18:1n-9). PO was abundant in linolenic acid (LNA, C18:3n-3), and SO was rich in linoleic acid (LA, C18:2n-6). In addition, FO was abundant in HUFA like docosahexaenoic acid (DHA, C22:6n-3), arachidonic acid (ARA, C20:4n-6), and eicosapentaenoic acid (EPA, C20:5n-3). The eight dietary treatments were: FO group (FO and without PC), FO-PC group (FO and 3% PC), PO group (PO and without PC), PO-PC group (PO and 3% PC), SO group (SO and without PC), SO-PC group (SO and 3% PC), OO (OO and without PC) and OO-PC (OO and 3% PC, Table 1). The procedure and method of making experimental diets have been depicted in our past study [4].

### 2.2. Feeding Experiment and Sampling

The Ethics Committee of East China Normal University approved the feeding experiment. After being acclimatized, 960 healthy and vital crablets (4.19 ± 0.01 g) were placed into 32 polyethylene buckets (length × width × height = 82 cm × 62 cm × 58 cm) at random, and the volume of each bucket was 300 L. The present study contained eight experimental treatments, and each experimental treatment was designed into four replicates. Each replicate contained 30 crablets, and four tiles and five plastic pipes were added to each bucket to reduce cannibalism. The feeding daily ration was 4% body mass and hand-fed thrice daily (20% at 06:30, 20% at 16:00, and 60% at 21:00 h) during the 8-week feeding experiment. The natural photoperiod was used in the present study. Water change and refuse clear (excrement and uneaten feed) were performed once a day. The water used in the present study was fully aerated, and specific water quality parameters have been reported by Bu et al. [37].

After being fasted for 24 h at the end of the 8-week experiment, crablets in each bucket were counted and weighed to collect the survival and growth data. Whole body composition (total lipid, ash, crude protein, and moisture) was determined using five crablets from each bucket. Crablets reduced mobility by using ice anesthesia. Subsequently, two crablets from each bucket (treatment with a total of eight crablets) were anatomized to obtain muscle and hepatopancreas samples, and the samples were stored at −80°C for further use after being quickly frozen with liquid nitrogen. The fatty acid composition of muscle and hepatopancreas was detected with six crablets, and eight crablets were used to determine the total lipid content of hepatopancreas and muscles.

### 2.3. Chemical Composition and Biochemical Analysis

The chloroform-methanol method reported by Bligh and Dyer [38] was used to analyze the total lipid content of hepatopancreas, muscles, diets, and whole crabs. Standard methods in the Association of Official Analytical Chemists [39] were also applied to detect the ash, moisture, and protein contents in diets and whole crabs. Detailed procedures have been given in our past study [4]. The fatty acid composition of diets, hepatopancreas, and muscle was detected by gas–chromatography (GC2010-plus, Shimadzu, Japan). The specific parameters of gas chromatography (nitrogen and oven temperature, chromatography column, injector, and detector temperature) and preparation of fatty acid methyl esters were the same as in our previous study [5]. The dietary fatty acid composition is shown in Table 2. The glutathione peroxidase (GSH-Px), total antioxidant capacity (T-AOC), and superoxide dismutase (SOD) activities, as well as the malondialdehyde (MDA) content in the hepatopancreas, were analyzed using commercial kits producing by Nanjing Jiancheng Bioengineering Institute.

### 2.4. Quantitative Real-Time PCR (qRT-PCR)

qRT-PCR was applied to determine the transcriptional levels of genes in hepatopancreas, including elongase of very long-chain fatty acids 6 (*elovl6*), lysophosphatidylcholine acyltransferase (*lpcat*), fatty acids synthase (*fas*), carnitine palmitoyl transferase (*cpt*), and sterol-regulatory element binding protein 1 (*srebp-1*). The RNA extraction, reverse transcription, reaction system, and amplification procedure of qRT-PCR were consistent with our past studies [28, 33]. In brief, total RNA was obtained by RNAiso plus (Takara, China), and FastKing RT kit (with gDNase) produced by Tiangen was applied to synthesize cDNA templates by using 1000 ng high-quality total RNA. qRT-PCR was performed in the CFX96 RT-PCR system with the reagent of ChamQ Universal SYBR qPCR Master Mix (Q711–02/03) purchased from Vazyme (Nanjing, China). Operating steps were followed by instructions of the commercial kit. The primer sequences of target genes and reference genes (Ubiquitin/ribosomal S27 fusion protein and *β*-actin) are given in Table 3. Equation *E* = 10 ^(−1/Slope)^ – 1 was used to calculate the amplification efficiency of primers, and the amplification efficiency was between 90% and 110% in the present study. The transcriptional levels of target genes in the OO group (OO and without PC) were chosen as the calibrator, and the expression mRNA levels of target genes were determined by using the 2^−*ΔΔ*Ct^ formula reported by Livak and Schmittgen [40].

### 2.5. Calculations and Statistical Analysis

The calculation formulas of survival (%), feed conversion ratio (FCR), specific growth rate (SGR, % day^−1^), and weight gain (WG, %) have been given in our previous studies [28, 33]. SPSS software (version 20.0) was used for all statistical analyses, and the results were given as means ± standard error. Statistical significance was set as a *p*-value less than 0.05. The normality and homoscedasticity of experimental data were checked prior to statistical analysis. Two-way ANOVA was chosen to analyze the main effects and any possible interactions between oil sources and PC levels. When the *p*-value was less than 0.05, One-way ANOVA followed by Duncan's multiple comparison tests was selected to investigate the significant differences among crabs fed the diets supplemented with different oil sources under the same PC level. In addition, the significant differences between the PC deficiency and addition groups were determined by the independent-sample *T*-test under the same oil source.

## 3. Results

### 3.1. Growth Performance, Feed Utilization, and Survival

**Figure 1:**
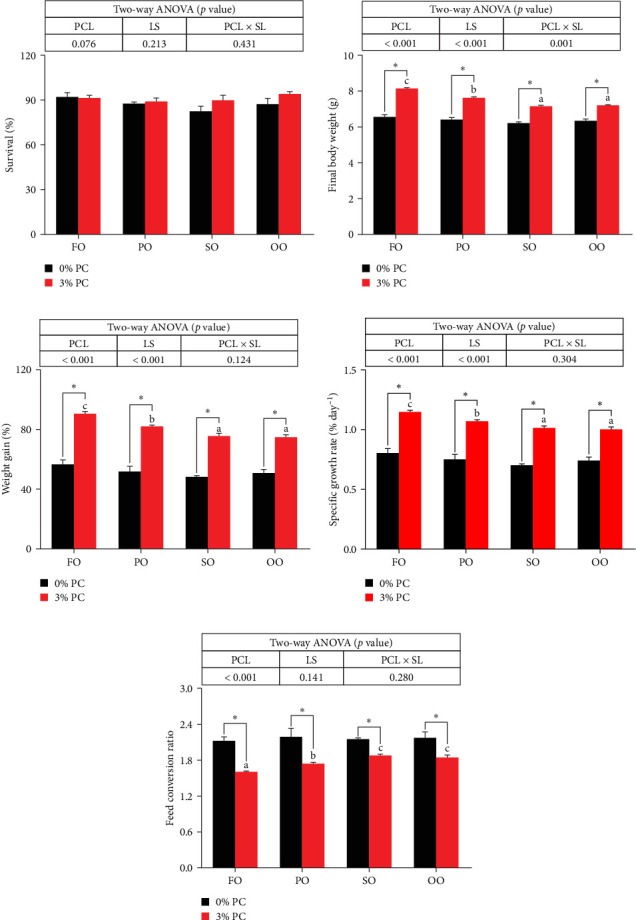
Growth performance and feed utilization of juvenile *E. sinensis* fed different experimental diets. (A) Survival, (B) final body weight, (C) weight gain, (D) specific growth rate, and (E) feed conversion ratio. Data are expressed as mean ± SEM (standard error of the mean) (*n* = 4). Columns with different superscripts are significantly different (*p* < 0.05). Capital letters indicate significant differences among crabs-fed diets supplemented with different LSs when the PCL was 0% (*p* < 0.05). Lowercase letters indicate significant differences among crabs fed diets supplemented with different LSs when the PCL was 3% (*p* < 0.05). In addition, *⁣*^*∗*^ indicates a significant difference between PCLs within the same LS (*p* < 0.05). FO, fish oil; LS, lipid source; OO, olive oil; PCL × SL, phosphatidylcholine level × lipid source; PCL, phosphatidylcholine level; PO, perilla oil; SO, safflower oil.

Final body weight (FBW) was significantly influenced by the dietary PC level, oil source, and their interaction (*p* < 0.05), while SGR and WG were only impacted by the dietary PC level and oil source (*p* < 0.05). The 3% PC-added groups showed dramatically higher SGR, FBW, and WG than PC-devoid groups under the same oil source condition (*p* < 0.05). Under the 3% PC condition, the FO-added group exhibited the highest values of SGR, FBW, and WG among all groups (*p* < 0.05), and the PO-added group also exhibited better growth-enhancing effects than OO-added and SO-added group (*p* < 0.05). However, there were no significant differences in SGR, FBW, and WG in different oil sources under PC-devoid conditions (*p* > 0.05). FCR was markedly affected by dietary PC levels, and the FCR in 3% of PC-added groups was significantly lower than in PC-devoid groups under the same oil source condition (*p* < 0.05). Besides, compared to the OO and SO groups, the FCR was markedly decreased in FO and SO groups, especially the FO group, when the dietary PC level was 3% (*p* < 0.05). The survival of crabs was not significantly impacted by dietary PC levels, oil sources, and their interaction (*p* > 0.05) (Figure 1).

### 3.2. Chemical Composition of the Whole Crab

**Figure 2:**
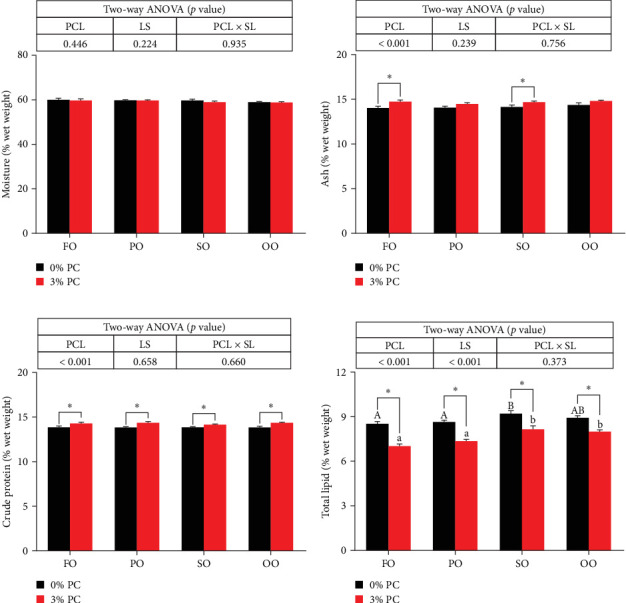
Proximate composition of juvenile *E. sinensis* (% wet weight) fed different experimental diets. (A) Moisture of the whole body, (B) ash of the whole body, (C) crude protein of the whole body, and (D) total lipid of the whole body. Data are expressed as mean ± SEM (standard error of the mean) (*n* = 4). Columns with different superscripts are significantly different (*p* < 0.05). Capital letters indicate significant differences among crabs-fed diets supplemented with different LSs when the PCL was 0% (*p* < 0.05). Lowercase letters indicate significant differences among crabs fed diets supplemented with different LSs when the PCL was 3% (*p* < 0.05). In addition, *⁣*^*∗*^ indicates a significant difference between PCLs within the same LS (*p* < 0.05). FO, fish oil; LS, lipid source; OO, olive oil; PCL × SL, phosphatidylcholine level × lipid source; PCL, phosphatidylcholine level; PO, perilla oil; SO, safflower oil.

Dietary PC levels notably affected the ash and crude protein contents of the whole crab (*p* < 0.05). Crude protein contents of whole crab in 3% PC-added groups were significantly higher than PC-devoid groups under the same oil source condition (*p* < 0.05). When dietary oil sources were SO and FO, 3% PC-added groups exhibited significantly higher values of whole-body ash contents than the PC-devoid groups (*p* < 0.05). Dietary PC levels and oil sources significantly impacted the total lipid contents of whole crab (*p* < 0.05). Under the same oil source condition, the total lipid contents of the whole crab in 3% PC-added groups were markedly lower than in PC-devoid groups (*p* < 0.05). Compared with the SO group, the total lipid contents of the whole crab were notably reduced in PO and FO groups when dietary PC was devoid (*p* < 0.05). In addition, under 3% PC-added condition, FO and PO groups exhibited markedly lower total lipid contents of the whole crab than OO and SO groups (*p* < 0.05). The moisture content of the whole crab was not significantly influenced by dietary PC levels, oil sources, and their interaction (*p* > 0.05) (Figure 2).

### 3.3. Antioxidative Capacity of Hepatopancreas

**Figure 3:**
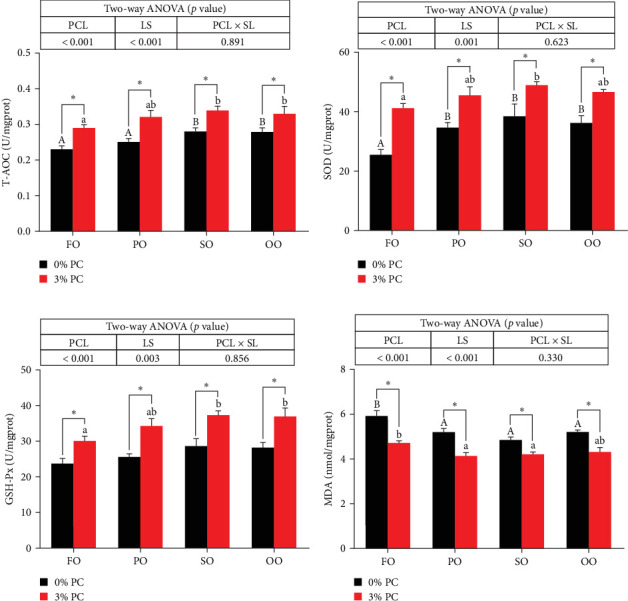
The activities of total antioxidant capacity (A), superoxide dismutase (B), glutathione peroxidase (C), and the content of malondialdehyde (D) in the hepatopancreas of juvenile *E. sinensis* fed different experimental diets. Data are expressed as mean ± SEM (standard error of the mean) (*n* = 8). Columns with different superscripts are significantly different (*p* < 0.05). Capital letters indicate significant differences among crabs-fed diets supplemented with different LSs when the PCL was 0% (*p* < 0.05). Lowercase letters indicate significant differences among crabs fed diets supplemented with different LSs when the PCL was 3% (*p* < 0.05). In addition, *⁣*^*∗*^ indicates a significant difference between PCLs within the same LS (*p* < 0.05). FO, fish oil; LS, lipid source; OO, olive oil; PCL × SL, phosphatidylcholine level × lipid source; PCL, phosphatidylcholine level; PO, perilla oil; SO, safflower oil.

GSH-Px, SOD, and T-AOC activities and MDA contents of hepatopancreas were notably affected by dietary PC and oil sources (*p* < 0.05). Compared with PC-devoid groups, 3% of PC-added groups showed markedly higher GSH-Px, SOD, and T-AOC activities of hepatopancreas under the same oil source condition (*p* < 0.05). T-AOC and GSH-Px activities of hepatopancreas in the FO group were significantly lower than in the SO and OO group when the dietary PC level was 3% (*p* < 0.05). In addition, under PC-devoid conditions, SO and OO groups had notably higher SOD and T-AOC activities in hepatopancreas than the FO group (*p* < 0.05). The 3% PC-added groups showed markedly lower MDA contents of hepatopancreas than PC-devoid groups under the same LS (*p* < 0.05). Compared with SO, PO, and OO groups, the MDA content in the FO group was significantly increased when dietary PC was devoid (*p* < 0.05). In addition, under 3% PC-added condition, MDA contents in the hepatopancreas of the SO and PO groups were markedly lower than those of the FO group (*p* < 0.05) (Figure 3).

### 3.4. Lipid Content and Gene Expression Levels in Hepatopancreas

**Figure 4:**
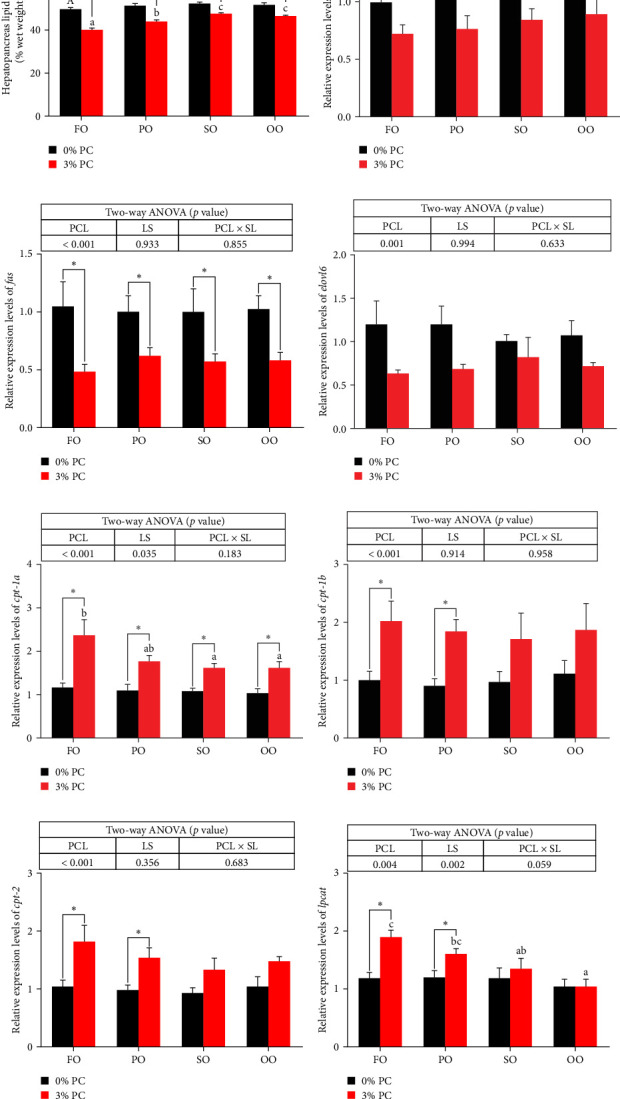
Lipid content (% wet weight) (A) and gene expression levels related to lipid metabolism (B–H) in the hepatopancreas of juvenile *E. sinensis* fed different experimental diets. Data are expressed as mean ± SEM (standard error of the mean) (*n* = 8). Columns with different superscripts are significantly different (*p* < 0.05). Capital letters indicate significant differences among crabs-fed diets supplemented with different LSs when the PCL was 0% (*p* < 0.05). Lowercase letters indicate significant differences among crabs-fed diets supplemented with different LSs when the PCL was 3% (*p* < 0.05). In addition, *⁣*^*∗*^ indicates a significant difference between PCLs within the same LS (*p* < 0.05). cpt, carnitine palmitoyl transferase; elovl6, elongase of very long chain fatty acids 6; fas, fatty acid synthase; FO, fish oil; lpcat, lysophosphatidylcholine acyltransferase; LS, lipid source; OO, olive oil; PCL × SL, phosphatidylcholine level × lipid source; PCL, phosphatidylcholine level; PO, perilla oil; SO, safflower oil; srebp-1, sterol-regulatory element binding protein 1.

The total lipid content of hepatopancreas was observably influenced by dietary PC levels, oil sources, and their interaction (*p* < 0.05). Compared with PC-devoid groups, the total lipid content of hepatopancreas was markedly decreased in 3% PC-added groups under the same oil source condition (*p* < 0.05). When the dietary PC level was 3%, the FO group exhibited the lowest value in total lipid content of hepatopancreas among all groups (*p* < 0.05), and the total lipid content of hepatopancreas in the PO group was also lower than OO and SO groups (*p* < 0.05). Transcriptional levels of *srebp-1*, *fas*, and *elovl6* (genes related to the synthesis of fatty acids) were markedly affected by dietary PC levels (*p* < 0.05). Compared with PC-devoid groups, expression levels of *elovl6* and *srebp-1* were downregulated in 3% of PC-added groups under the same oil source condition, although no significance was observed. In addition, diets supplemented with 3% PC notably suppressed *fas* expression compared to PC-devoid diets under the same oil source condition (*p* < 0.05). Expression levels of *cpt-1a*, *cpt-1b*, and *cpt-2* (genes involved in *β*-oxidation of fatty acids) were significantly affected by dietary PC levels (*p* < 0.05), and *cpt-1a* was also impacted by dietary oil sources (*p* < 0.05). The 3% PC-added groups showed higher transcriptional levels of *cpt-1a* than PC-devoid groups under the same oil source condition (*p* < 0.05), and *cpt-1a* expression levels were significantly upregulated in the FO group compared with SO and OO groups when dietary PC level was 3% (*p* < 0.05). Compared with PC-devoid groups, *cpt-1b* and *cpt-2* expression levels were increased in the 3% PC-added groups under the same oil source condition and when the dietary oil source was FO or PO, showing significant differences (*p* < 0.05). *lpcat* expression was markedly affected by dietary PC levels and oil sources (*p* < 0.05). The 3% PC-added groups exhibited significantly higher *lpcat* transcriptional levels than PC-devoid groups when the dietary LS was FO or PO (*p* < 0.05). The FO group exhibited the highest *lpcat* expression levels when the dietary PC level was 3%, showing significant differences between safflower and OO groups (*p* < 0.05). Besides, under the 3% PC-added condition, the PO group showed a significantly higher *lpcat* mRNA level than the OO group (*p* < 0.05), and there was no significant difference between FO and PO groups (*p* > 0.05) (Figure 4).

### 3.5. Composition of Fatty Acids in Hepatopancreas

Saturated fatty acids (SFAs) level in hepatopancreas was significantly impacted by dietary oil sources (*p* < 0.05), and the FO group showed markedly higher SFA levels than other oil sources groups (*p* < 0.05) in both 0% and 3% PC condition (*p* < 0.05). Dietary PC levels, oil sources, and their interaction dramatically affected the monounsaturated fatty acid (MUFA) level of the hepatopancreas (*p* < 0.05). Compared with other oil sources-added groups, the MUFA level of hepatopancreas was notably increased in OO groups whether the dietary PC level was 0% or 3% (*p* < 0.05). Except for FO, PC-devoid groups exhibited significantly lower MUFA levels of hepatopancreas than 3% PC-added groups under the same oil source condition (*p* < 0.05). OA (C18:1n-9) level of hepatopancreas was markedly influenced by dietary oil sources and the interaction of dietary PC levels and oil sources (*p* < 0.05), and the OO group showed a significantly higher OA (C18:1n-9) level of hepatopancreas than other oil sources groups in both 0% and 3% PC condition (*p* < 0.05). In addition, the PUFA, LNA (C18:3n-3), and LA (C18:2n-6) levels of hepatopancreas were notably affected by dietary PC levels, oil sources, and their interaction (*p* < 0.05). PO and SO groups had markedly higher PUFA levels of hepatopancreas than FO and OO groups, whether dietary PC level was 0% or 3% (*p* < 0.05). In both 0% and 3% PC conditions, the highest LA (C18:2n-6) and LNA (C18:3n-3) levels of hepatopancreas were found in the SO group and PO group, respectively (*p* < 0.05). In addition, there was a significant effect of dietary oil sources on HUFA, EPA (C20:5n-3), ARA (C20:4n-6), and DHA (C22:6n-3) levels, as well as n-6 fatty acids/n-3 fatty acids value in hepatopancreas (*p* < 0.05). The FO group showed notably higher EPA (C20:5n-3), ARA (C20:4n-6), HUFA, and DHA (C22:6n-3) levels of hepatopancreas than other oil sources groups in both 0% and 3% PC conditions (*p* < 0.05). n-6 fatty acids/n-3 fatty acids value was significantly reduced in perilla and FO groups compared with safflower and OO groups, whether the dietary PC level was 0% or 3% (*p* < 0.05) (Table 4).

### 3.6. Composition of Fatty Acids in Muscle

SFA level in muscle was significantly influenced by dietary oil sources (*p* < 0.05), and the FO group showed significantly higher SFA levels than other oil sources groups in both 0% and 3% PC conditions (*p* < 0.05). Dietary oil sources notably impacted OA (C18:1n-9) and MUFA levels of muscle and the interaction of dietary PC levels and oil sources (*p* < 0.05), and OO groups exhibited significantly higher MUFA and OA (C18:1n-9) levels of muscle than other oil sources groups whether the dietary PC level was 0% or 3% (*p* < 0.05). In addition, dietary oil sources and the interaction of dietary PC levels and oil sources observably affected LA (C18:2n-6) level of muscle (*p* < 0.05), and PUFA and LNA (C18:3n-3) levels of muscle were dramatically impacted by dietary oil sources (*p* < 0.05). In both 0% and 3% PC conditions, FO and OO groups showed prominently lower PUFA levels in muscle than PO and SO groups (*p* < 0.05). SO and PO exhibited the highest LA (C18:2n-6) and (C18:3n-3) levels of muscle, respectively, whether the dietary PC level was 0% or 3% (*p* < 0.05). Dietary oil sources significantly impacted HUFA and EPA (C20:5n-3) levels of muscle and the interaction of dietary PC levels and oil sources (*p* < 0.05), while DHA (C22:6n-3) and ARA (C20:4n-6) levels of muscle were only markedly affected by dietary oil sources (*p* < 0.05). The FO group had observably higher DHA (C22:6n-3), ARA (C20:4n-6), HUFA, and EPA (C20:5n-3) levels of muscle than other oil sources groups in both 0% and 3% PC condition (*p* < 0.05). Besides, dietary oil sources and the interaction of dietary oil sources and PC levels significantly affected the n-6 fatty acids/n-3 fatty acids value of muscle (*p* < 0.05), and compared with SO and OO groups, n-6 fatty acids/n-3 fatty acids value of muscle was markedly reduced in FO and PO groups (*p* < 0.05) (Table 5).

## 4. Discussion

In the present study, dietary PC markedly promoted the growth and feed utilization of juvenile *E. sinensis*. These results are consistent with past findings in *Oreochromis niloticus* [41] and *Portunus trituberculatus* [42]. Similar results were also discovered in our previous studies on the same species [4, 28], which further supports the results of the present study. The positive functions of dietary PC may be associated with its roles in improving diet quality, providing essential nutrients, and optimizing lipid metabolism [1]. The growth performance between vegetable oils and FO groups did not show a significant difference under the PC-devoid condition. Likewise, Han et al. [43] found that juvenile *P. trituberculatus* fed FO did not show higher WG than those fed diets with vegetable oils (linseed oil, rapeseed oil, and soybean oil). In addition, PO and FO groups showed better growth-promoting effects than SO and OO groups under the 3% PC-added condition, especially FO. Similarly, González-Félix et al. [34] reported that menhaden oil was more beneficial for the growth of *L. vannamei* than vegetable oil in the presence of phospholipids. Besides, Bu et al. [44] reported that PO showed more growth-enhancing effects than other vegetable oils (palm oil, OO, and SO). The possible reason is that the endogenous synthesis of HUFA is very limited, and LNA (C18:3n-3) is an important essential fatty acid for *E. sinensis*. Furthermore, dietary PC and FO or rapeseed oil may form a new physiological-active phospholipids via biochemical reactions and avoid the oxidation of FO or rapeseed oil through antioxidant action, ultimately promoting growth.

Previous studies have indicated that the whole-body lipid contents of aquatic animals were markedly affected by dietary LSs [45–47]. The present study found that compared with SO and OO groups, the total lipid contents of whole crab were reduced in FO and PO groups, indicating that diets enriched in n-3 PUFA or HUFA could inhibit lipid accumulation in the whole body. Likewise, Guo et al. [47] reported that juvenile golden pompano (*Trachinotus ovatus*) fed a diet added with FO showed the lowest whole-body lipid contents. Besides, dietary PC levels markedly affected the total lipid contents of the whole crab in the present study, and compared with PC-devoid groups, the total lipid contents were observably reduced in PC-added groups. The lipid-lowering effects of phospholipids were consistently found in teleost fish [32, 48]. Based on previous studies, the possible lipid-lowering mechanism of dietary PC may relate to its roles in suppressing triglyceride synthesis and promoting triglyceride transport and oxidation [28, 32]. Besides lipid contents, dietary PC levels also impacted the protein contents of the whole crab, and the PC-added groups showed higher protein contents than the PC-devoid groups, indicating that dietary phospholipids could facilitate whole-body protein accumulation. This result agrees with the findings in *Megalobrama amblycephala* [48], *Seriola dumerili* [49], *Larimichthys crocea* [50], and *E. sinensis* [28]. The positive effects of dietary PC in protein deposition may be connected with its roles in accelerating lipid utilization and reducing protein consumption as an energy source (protein-sparing effect).

Generally, the antioxidant enzyme activities and contents of lipid peroxidation metabolite (MDA) were used to evaluate the antioxidant status and oxidative damage degree for organisms [48, 51]. Many previous studies showed that lipid peroxidation occurred closely with the degree of unsaturation of fatty acids, and high levels of HUFA were easier to induce lipid peroxidation [52, 53]. Consistently, the present study found that the SOD and T-AOC activities of the hepatopancreas were markedly decreased, and MDA contents were significantly increased in FO groups compared with vegetable oil groups, suggesting that high HUFA levels in diets could enhance the burden of the antioxidant system and the risk of lipid peroxidation in juvenile *E. sinensis*. Similarly, Yuan et al. [54] reported that high HUFA levels in diets were the main inducement of lipid peroxidation for juvenile *P. trituberculatus*, further supporting the present results. On the contrary, studies in *O*. *niloticus* [12], *Seriola lalandi* [55], *Lateolabrax japonicas* [15], and *L. crocea* [14] indicate that excessive dietary FO replaced by vegetable oils can cause antioxidant capacity reduction. The discrepancy may be related to different nutritional conditions and animal species. In addition, past studies have manifested that dietary phospholipids were beneficial for the antioxidant status of aquatic animals [27, 56]. Uniformly, the PC effectively alleviated oxidative stress and lipid peroxidation caused by HUFA in the present study. The antioxidant mechanism of phospholipids is still ill-defined, which may be related to the functional groups of the phospholipids like choline, ethanolamine, inositol, and hydroxyl [1]. The results above suggest that dietary PC can relieve the adverse effects of high HUFA levels in diets by improving the body's antioxidant capacity.

Previous studies have indicated that the compositions of fatty acids were closely related between diets and tissues in aquatic animals like *P. trituberculatus* [26], *Scylla paramamosain* [57], *L. vannamei* [58], and *Penaeus monodon* [35]. Similar results were also observed in the present study, which found that compositions of fatty acids in muscle and hepatopancreas were positively associated with that in diets. In addition to the compositions of fatty acids, the total lipid contents of the hepatopancreas were also impacted by dietary treatments, and the total lipid contents were significantly reduced in PC-added groups compared with PC-devoid groups. This result aligns with our past findings in the same species [28, 33]. The lipid-lowering roles of phospholipids were also observed in mammals and teleost fish like mice [22, 23], *O. niloticus* [41], and *L. crocea* [32]. Past studies have indicated that the lipid-lowering mechanism of phospholipids is closely associated with its function in promoting lipid utilization and inhibiting lipid synthesis [22, 23, 28, 32, 33]. Likewise, the diets added with 3% PC significantly suppressed the gene expression related to lipid synthesis and facilitated the gene expression involved in lipid oxidation in the present study. Generally, the sn-2 site of phospholipids (including PC) molecules tended to connect PUFA, while the sn-1 position preferentially with SFA and MUFA [25, 59]. Studies have suggested that the sn-2 site of phospholipids lined with different fatty acids showed different lipid metabolism patterns, especially in connection with n-3 PUFA, and possessed better lipid-lowering effects through promoting lipid utilization [4, 21–23]. For example, Liu et al. [22] found that compared with soy-derived phospholipids, EPA-phospholipids were more effective in decreasing triglyceride in the liver and serum by promoting lipid *β*-oxidation and inhibiting lipid synthesis. Similarly, Liu et al. [23] reported that EPA-PC could alleviate lipid deposition more than LA-PC for orotic acid-induced nonalcoholic fatty liver. Fatty acid changes at sn-2 sites of PC molecules are mainly involved in the PC remodeling reaction, and two enzymes (LPCAT and phospholipase A_2_) participate in this process, especially LPCAT is responsible for rebinding fatty acids with appropriate saturation and chain length to the lysophosphatidylcholine, finally producing new PC [60]. Wang et al. [25] have indicated that glycerophospholipids (GPs) are the primary lipid molecules and are impacted by dietary DHA (C22:6n-3)/EPA (C20:5n-3) ratios in the hepatopancreas of *S. Paramemosain* through lipidomic analysis. The optimal dietary DHA (C22:6n-3)/EPA (C20:5n-3) ratios (2.3) can promote more EPA (C20:5n-3), ARA (C20:4n-6), and DHA (C22:6n-3) and combine to the sn-2 location of PC molecules. Likewise, Xu et al. [59] found that the fatty acid compositions of PC and PE were positively correlated with the fatty acid compositions of diets in *E. sinensis*. These results suggest that dietary fatty acids can change the fatty acid compositions of phospholipids (including PC). The present study shows that under 3% PC-added condition, FO and PO possess more lipid-decreasing functions than SO and OO, especially FO. We further detected the key gene expression levels involved in PC remodeling reaction, and the results showed that the FO and PO groups exhibited higher *lpcat* expression levels in hepatopancreas, especially the FO group, which may indicate that FO and PO could promote more n-3 PUFA acclimation in PC molecules. Combined with the gene expression levels of fatty acid *β*-oxidation and functions of HUFA-phospholipid, we assumed that the FO and PO may form new physiological-active PC contained n-3 PUFA with dietary PC through activating PC remodeling reaction and then promoting fatty acid utilization, finally reducing the lipid accumulation in hepatopancreas.

In conclusion, when PC was added to the feed, FO could significantly promote growth and feed utilization compared with other oil sources, and PO was better than SO and OO. The dietary PC contributed to the FO and PO showing more promoting-growth effects, probably by preventing the FO and PO from lipid peroxidation and forming physiological-active PC with the FO and PO to further improve lipid utilization. These results suggest that FO and PO were better oil sources for *E. sinensis* and provided the possibility of using PC supplementation to use oil sources efficiently in the crab diet.

## Figures and Tables

**Table 1:** Ingredient formulation (g/kg dry weight) and proximate composition (% dry weight) of the eight experimental diets fed to juvenile *E. sinensis*.

Ingredients	Experimental diets^1^							
	FO	FO-PC	PO	PO-PC	SO	SO-PC	OO	OO-PC
Casein	320	320	320	320	320	320	320	320
Gelatin	80	80	80	80	80	80	80	80
Corn starch	280	280	280	280	280	280	280	280
Vitamin premix^2^	30	30	30	30	30	30	30	30
Mineral premix^3^	20	20	20	20	20	20	20	20
Carboxymethyl cellulose	20	20	20	20	20	20	20	20
Betaine^4^	30	30	30	30	30	30	30	30
Cholesterol^4^	5	5	5	5	5	5	5	5
Butylated hydroxytoluene^4^	1	1	1	1	1	1	1	1
Choline chloride^4^	5	5	5	5	5	5	5	5
Cellulose	124	124	124	124	124	124	124	124
Fish oil	55	55	—	—	—	—	—	—
Perilla oil	—	—	55	55	—	—	—	—
Safflower oil	—	—	—	—	55	55	—	—
Olive oil	—	—	—	—	—	—	55	55
Soybean oil	30	—	30	—	30	—	30	—
Phosphatidylcholine^5^	—	30	—	30	—	30	—	30
Total	1000	1000	1000	1000	1000	1000	1000	1000
Proximate composition								
Moisture	9.46	9.63	9.89	9.69	9.51	9.37	9.48	9.41
Crude protein	35.96	36.23	36.30	36.25	36.13	36.10	36.06	36.32
Total lipid	9.18	9.27	9.57	9.18	9.36	9.46	9.30	9.22
Ash	3.14	3.27	3.11	3.32	3.16	3.34	3.14	3.29
Phosphatidylcholine	0.00	2.79	0.00	2.74	0.00	2.72	0.00	2.80

^1^FO, fish oil and without phosphatidylcholine; FO-PC, fish oil and 3% phosphatidylcholine; PO, perilla oil and without phosphatidylcholine; PO-PC, perilla oil and 3% phosphatidylcholine; SO, safflower oil and without phosphatidylcholine; SO-PC, safflower oil and 3% phosphatidylcholine; OO, olive oil and without phosphatidylcholine; OO-PC, olive oil and 3% phosphatidylcholine.

^2^Vitamin premix (per 100 g premix): retinol acetate, 0.043 g; thiamin hydrochloride, 0.15 g; riboflavin, 0.0625 g; Ca pantothenate, 0.3 g; niacin, 0.3 g; pyridoxine hydrochloride, 0.225 g; para-aminobenzoic acid, 0.1 g; ascorbic acid, 0.5 g; biotin, 0.005 g; folic acid, 0.025 g; cholecalciferol, 0.0075 g; *α*-tocopherol acetate, 0.5 g; menadione, 0.05 g; inositol, 1 g. All ingredients are filled with *α*-cellulose to 100 g.

^3^Mineral premix (per 100 g premix): KH_2_PO_4_, 21.5 g; NaH_2_PO_4_, 10.0 g; Ca (H_2_PO_4_)_2_, 26.5 g; CaCO_3_, 10.5 g; KCl, 2.8 g; MgSO_4_·7H_2_O, 10.0 g; AlCl_3_·6H_2_O, 0.024 g; ZnSO_4_·7H_2_O, 0.476 g; MnSO_4_·6H_2_O, 0.143 g; KI, 0.023 g; CuCl_2_·2H_2_O, 0.015 g; CoCl_2_·6H_2_O, 0.14 g; Calcium lactate, 16.50 g; Fe-citrate, 1 g. All ingredients are diluted with *α*-cellulose to100 g.

^4^Sangon Biotech, Ltd., Shanghai, China.

^5^Shanghai Taiwei, Ltd., Shanghai, China.

**Table 2:** Fatty acid composition (% total fatty acids) of eight experimental diets.

Fatty acids	Experimental diets^1^							
	FO	FO-PC	PO	PO-PC	SO	SO-PC	OO	OO-PC
C14:0	4.58	4.85	0.39	0.45	0.47	0.54	0.41	0.47
C14:1	0.16	0.17	0.05	0.06	0.05	0.06	0.05	0.06
C15:0	0.71	0.75	0.08	0.09	0.08	0.10	0.07	0.09
C15:1	0.01	0.02	0.00	0.00	0.01	0.00	0.01	0.00
C16:0	19.28	19.48	8.96	8.68	9.45	9.46	14.66	15.03
C16:1	3.52	3.84	0.13	0.20	0.14	0.14	0.68	0.65
C17:0	0.52	0.54	0.06	0.05	0.04	0.06	0.06	0.09
C17:1	0.29	0.32	0.05	0.05	0.05	0.05	0.08	0.10
C18:0	4.51	4.53	1.57	1.15	1.58	1.22	1.74	1.50
C18:1n-9	18.61	15.29	17.30	13.49	17.06	14.06	53.30	53.37
C18:2n-6	22.70	23.81	26.57	28.18	67.18	71.51	25.02	25.21
C18:3n-6	0.27	0.08	0.32	0.09	0.17	0.01	0.18	0.01
C20:1	0.10	0.07	0.06	0.04	0.03	0.02	0.06	0.03
C18:3n-3	4.23	4.06	44.19	47.23	3.28	2.47	3.41	3.21
C22:0	1.42	1.52	0.04	0.02	0.08	0.08	0.05	0.04
C20:3n-6	0.18	0.18	0.03	0.04	0.02	0.03	0.03	0.03
C20:3n-3	0.81	0.77	0.08	0.04	0.22	0.15	0.17	0.08
C20:4n-6	3.76	4.04	0.04	0.02	0.01	0.01	0.00	0.01
C20:5n-3	5.19	5.62	0.04	0.06	0.04	0.04	0.01	0.02
C22:6n-3	9.16	10.05	0.05	0.07	0.03	0.01	0.01	0.01
SFA^2^	31.02	31.68	11.09	10.45	11.71	11.45	16.99	17.21
MUFA^3^	22.69	19.71	17.60	13.84	17.34	14.34	54.18	54.22
PUFA^4^	27.20	27.95	71.08	75.49	70.63	73.99	28.61	28.43
HUFA^5^	19.09	20.66	0.23	0.22	0.33	0.23	0.22	0.14
n-3 FA^6^	19.39	20.50	44.35	47.39	3.57	2.66	3.60	3.32
n-6 FA^7^	26.90	28.12	26.95	28.32	67.38	71.56	25.23	25.26
n-6/n-3	1.39	1.37	0.61	0.60	18.86	26.89	7.01	7.61

^1^FO, fish oil and without phosphatidylcholine; FO-PC, fish oil and 3% phosphatidylcholine; PO, perilla oil and without phosphatidylcholine; PO-PC, perilla oil and 3% phosphatidylcholine; SO, safflower oil and without phosphatidylcholine; SO-PC, safflower oil and 3% phosphatidylcholine; OO, olive oil and without phosphatidylcholine; OO-PC, olive oil and 3% phosphatidylcholine.

^2^SFA (saturated fatty acid): C14:0, C15:0, C16:0, C17:0, C18:0, C22:0.

^3^MUFA (monounsaturated fatty acid): C14:1, C15:1, C16:1, C17:1, C18:1n-9, C20:1.

^4^PUFA (polyunsaturated fatty acid): C18:2n-6, C18:3n-6, C18:3n-3.

^5^HUFA (highly unsaturated fatty acid): C20:3n-6, C20:3n-3, C20:4n-6, C20:5n-3, C22:6n-3.

^6^n-3 FA (n-3 fatty acid): C18:3n-3, C20:3n-3, C20:5n-3, C22:6n-3.

^7^n-6 FA (n-3 fatty acid): C18:2n-6, C18:3n-6, C20:3n-6, C20:4n-6.

**Table 3:** Primer pair sequences and product size of the genes used for quantitative real-time PCR.

Gene	Position	Primer sequence	Length	Tm	Product size (bp)
*srebp-1*	Forward	TCTTCACACCCTCTGGACGC	20	60.5	162
	Reverse	CCAAGGTTGTAATGGCACGC	20	61.3	
*fas*	Forward	GTCCCTTCTTCTACGCCATCC	21	60.3	127
	Reverse	CGCTCTCCAGGTCAATCTTCAC	22	61.3	
*elovl6*	Forward	TGAGAAGCGGCAATGGATGAAG	22	64.1	164
	Reverse	TGGAGAAGAGGGCCAGGAAGAC	22	64.0	
*cpt-1a*	Forward	CATCTGGACACCCACCTCCA	20	60.8	183
	Reverse	ATCTCCTCACCCGGCACTCT	20	60.7	
*cpt-1b*	Forward	GGCATTCTCCTTTGCCATCAC	21	61.8	138
	Reverse	ACACCACACCGCACATTGTTC	21	61.2	
*cpt-2*	Forward	AGCAGGCAGTGGCTCAGTTTA	21	60.2	169
	Reverse	AAGGCAAGGAAGGGGTTGTAG	21	60.1	
*lpcat*	Forward	AAAGGTCAGCAGGCACCTCCA	21	64.3	188
	Reverse	TCCTCTCGCCACACATACACAGG	23	64.6	
*β-Actin*	Forward	TCGTGCGAGACATCAAGGAAA	21	61.5	178
	Reverse	AGGAAGGAAGGCTGGAAGAGTG	22	61.6	
*S27*	Forward	CCCCCAAGAAGATCAAGCACA	21	62.3	179
	Reverse	CAGATGGCAGCGACCACAGTA	21	61.8	

*Note:* Transfer protein; *S27*, ubiquitin/ribosomal S27 fusion protein.

Abbreviations: *cpt-1a*, carnitine palmitoyl transferase 1a; *cpt-1b*, carnitine palmitoyl transferase 1b; *cpt-2*, carnitine palmitoyl transferase 2; *elovl6*, elongase of very long-chain fatty acids 6; *fas*, fatty acids synthase; *lpcat*, lysophosphatidylcholine acyltransferase; *srebp-1*, sterol-regulatory element binding protein 1.

**Table 4:** Fatty acid composition (% total fatty acids) in the hepatopancreas of juvenile *E. sinensis* fed different experimental diets.

Fatty acids	Experimental diets^1^								Two-way ANOVA (*p* value)^2^		
	FO	FO-PC	PO	PO-PC	SO	SO-PC	OO	OO-PC	PCL	LS	PCL × SL
C14:0	2.18 ± 0.06^c^	2.25 ± 0.05^b^	0.79 ± 0.01^b^	0.81 ± 0.01^a^	0.76 ± 0.01^ab^	0.84 ± 0.04^a^	0.70 ± 0.01^a^	0.75 ± 0.01^a^	0.025	<0.001	0.805
C14:1	0.29 ± 0.01^c^	0.33 ± 0.01^b^	0.17 ± 0.01^b^	0.17 ± 0.02^a^	0.09 ± 0.00^A,a^	0.15 ± 0.01^B,a^	0.15 ± 0.00^A,b^	0.17 ± 0.01^B,a^	<0.001	<0.001	0.127
C15:0	0.48 ± 0.01^b^	0.48 ± 0.01^b^	0.23 ± 0.02^a^	0.26 ± 0.02^a^	0.25 ± 0.01^a^	0.26 ± 0.02^a^	0.23 ± 0.01^a^	0.26 ± 0.03^a^	0.186	<0.001	0.824
C15:1	0.04 ± 0.01^c^	0.04 ± 0.00^c^	0.04 ± 0.01^bc^	0.03 ± 0.00^b^	0.02 ± 0.00^a^	0.02 ± 0.00^a^	0.03 ± 0.00^ab^	0.03 ± 0.00^ab^	0.690	<0.001	0.939
C16:0	19.41 ± 0.32^b^	19.96 ± 0.33^b^	16.91 ± 0.38^A,a^	18.25 ± 0.25^B,a^	17.65 ± 0.22^a^	19.26 ± 0.83^ab^	17.38 ± 0.25^A,a^	18.21 ± 0.12^B,a^	0.001	<0.001	0.544
C16:1	13.84 ± 0.10^d^	14.53 ± 0.43^b^	9.82 ± 0.36^A,b^	12.48 ± 0.50^B,a^	8.80 ± 0.11^A,a^	12.52 ± 0.67^B,a^	11.84 ± 0.33^A,c^	14.13 ± 0.62^B,ab^	<0.001	<0.001	0.020
C17:0	0.27 ± 0.01^b^	0.27 ± 0.01^b^	0.10 ± 0.01^a^	0.11 ± 0.01^a^	0.12 ± 0.01^a^	0.10 ± 0.01^a^	0.11 ± 0.01^B,a^	0.08 ± 0.01^A,a^	0.045	<0.001	0.163
C17:1	0.63 ± 0.02^b^	0.65 ± 0.02^b^	0.30 ± 0.02^a^	0.36 ± 0.03^a^	0.32 ± 0.01^a^	0.36 ± 0.01^a^	0.35 ± 0.02^a^	0.38 ± 0.03^a^	0.026	<0.001	0.873
C18:0	2.23 ± 0.04^b^	2.05 ± 0.08^b^	2.17 ± 0.06^B,b^	1.90 ± 0.06^A,ab^	2.50 ± 0.02^B,c^	1.98 ± 0.07^A,b^	2.01 ± 0.04^B,a^	1.74 ± 0.07^A,a^	<0.001	<0.001	0.045
C18:1n-9	30.88 ± 0.53^c^	29.98 ± 0.15^b^	28.59 ± 0.30^b^	29.18 ± 0.27^b^	26.48 ± 0.23^A,a^	27.39 ± 0.18^B,a^	47.70 ± 0.39^d^	46.01 ± 0.68^c^	0.342	<0.001	0.008
C18:2n-6	15.70 ± 0.35^a^	14.45 ± 0.55^ab^	18.26 ± 0.29^B,b^	15.81 ± 0.65^A,b^	38.02 ± 0.16^B,c^	33.90 ± 0.54^A,c^	15.11 ± 0.47^B,a^	13.39 ± 0.39^A,a^	<0.001	<0.001	0.014
C18:3n-6	0.27 ± 0.01^B,c^	0.17 ± 0.01^A,c^	0.27 ± 0.01^Bc^	0.13 ± 0.00^A,b^	0.25 ± 0.00^B,b^	0.11 ± 0.00^A,a^	0.22 ± 0.00^B,a^	0.10 ± 0.01^A,a^	<0.001	<0.001	0.002
C20:1	0.10 ± 0.00^B,b^	0.03 ± 0.00^A,ab^	0.04 ± 0.01^a^	0.05 ± 0.01^b^	0.09 ± 0.01^B,b^	0.03 ± 0.00^A,a^	0.09 ± 0.01^B,b^	0.03 ± 0.00^A,a^	<0.001	0.007	<0.001
C18:3n-3	4.18 ± 0.07^b^	4.25 ± 0.06^b^	19.70 ± 0.77^c^	17.41 ± 0.49^c^	2.40 ± 0.10^a^	2.34 ± 0.11^a^	2.50 ± 0.09^a^	2.32 ± 0.02^a^	0.013	<0.001	0.004
C22:0	0.41 ± 0.02^b^	0.42 ± 0.01^b^	0.05 ± 0.01^a^	0.05 ± 0.00^a^	0.06 ± 0.00^a^	0.06 ± 0.00^a^	0.05 ± 0.00^a^	0.05 ± 0.00^a^	0.788	<0.001	0.596
C20:3n-6	0.59 ± 0.02^A,b^	0.67 ± 0.02^B,a^	0.69 ± 0.04^c^	0.79 ± 0.05^a^	1.01 ± 0.02^A,d^	1.20 ± 0.07^B,b^	0.52 ± 0.01^A,a^	0.63 ± 0.03^B,a^	<0.001	<0.001	0.507
C20:3n-3	1.09 ± 0.05^b^	1.20 ± 0.05^b^	1.19 ± 0.08^b^	1.09 ± 0.06^b^	0.15 ± 0.01^a^	0.15 ± 0.01^a^	0.13 ± 0.01^a^	0.13 ± 0.01^a^	0.901	<0.001	0.091
C20:4n-6	0.98 ± 0.09^b^	1.00 ± 0.05^b^	0.30 ± 0.03^a^	0.37 ± 0.05^a^	0.34 ± 0.03^a^	0.37 ± 0.01^a^	0.31 ± 0.01^A,a^	0.34 ± 0.01^B,a^	0.281	<0.001	0.961
C20:5n-3	2.75 ± 0.09^b^	2.73 ± 0.09^b^	0.39 ± 0.04^a^	0.52 ± 0.09^a^	0.45 ± 0.06^a^	0.48 ± 0.02^a^	0.35 ± 0.02^a^	0.39 ± 0.02^a^	0.400	<0.001	0.771
C22:6n-3	4.11 ± 0.09^b^	4.42 ± 0.14^b^	0.17 ± 0.02^a^	0.25 ± 0.03^a^	0.23 ± 0.01^a^	0.23 ± 0.02^a^	0.19 ± 0.01^a^	0.18 ± 0.01^a^	0.057	<0.001	0.074
SFA^3^	24.84 ± 0.45^c^	25.44 ± 0.37^b^	20.22 ± 0.40^a^	21.38 ± 0.26^a^	21.34 ± 0.24^b^	21.59 ± 0.27^a^	20.49 ± 0.24^ab^	21.08 ± 0.11^a^	0.007	<0.001	0.578
MUFA^4^	45.43 ± 0.38^c^	45.59 ± 0.46^c^	38.68 ± 0.78^A,b^	42.29 ± 0.61^B,b^	35.80 ± 0.17^A,a^	39.86 ± 0.38^B,a^	60.17 ± 0.52^A,d^	61.89 ± 0.29^B,d^	<0.001	<0.001	0.001
PUFA^5^	20.14 ± 0.40^b^	18.87 ± 0.58^b^	38.22 ± 1.00^B,c^	33.36 ± 0.74^A,c^	40.67 ± 0.22^B,d^	36.24 ± 0.54^A,d^	17.83 ± 0.55^B,a^	15.50 ± 0.32^A,a^	<0.001	<0.001	0.015
HUFA^6^	9.60 ± 0.23^d^	10.11 ± 0.24^d^	2.88 ± 0.22^c^	2.99 ± 0.19^c^	2.18 ± 0.12^b^	2.40 ± 0.12^b^	1.52 ± 0.07^a^	1.61 ± 0.03^a^	0.069	<0.001	0.592
n-3 FA^7^	12.13 ± 0.09^b^	12.60 ± 0.26^b^	21.48 ± 0.85^c^	19.24 ± 0.49^c^	3.23 ± 0.16^a^	3.22 ± 0.15^a^	3.17 ± 0.12^a^	3.00 ± 0.02^a^	0.077	<0.001	0.007
n-6 FA^8^	17.60 ± 0.45^b^	16.38 ± 0.64^b^	19.61 ± 0.34^B,c^	17.10 ± 0.74^A,b^	39.62 ± 0.19^B,d^	35.46 ± 0.55^A,c^	16.17 ± 0.51^B,a^	14.45 ± 0.42^A,a^	<0.001	<0.001	0.032
n-6/n-3	1.45 ± 0.04^a^	1.30 ± 0.06^a^	0.92 ± 0.03^a^	0.89 ± 0.05^a^	12.38 ± 0.58^c^	11.68 ± 0.41^c^	5.11 ± 0.10^B,b^	4.70 ± 0.11^A,b^	0.104	<0.001	0.619

*Note:* Data are expressed as mean ± SEM (standard error of the mean) (*n* = 6). Values in the same line with different superscripts are significantly different (*p* < 0.05). ^AB^ Indicates a significant difference between phosphatidylcholine levels within the same lipid source (*p* < 0.05). ^abc^ Indicates a significant difference between lipid sources within the same phosphatidylcholine level (*p* < 0.05).

^1^FO, fish oil and without phosphatidylcholine; FO-PC, fish oil and 3% phosphatidylcholine; PO, perilla oil and without phosphatidylcholine; PO-PC, perilla oil and 3% phosphatidylcholine; SO, safflower oil and without phosphatidylcholine; SO-PC, safflower oil and 3% phosphatidylcholine; OO, olive oil and without phosphatidylcholine; OO-PC, olive oil and 3% phosphatidylcholine.

^2^PCL, phosphatidylcholine level; LS, lipid source; PCL × SL, phosphatidylcholine level × lipid source.

^3^SFA (saturated fatty acid): C14:0, C15:0, C16:0, C17:0, C18:0, C22:0.

^4^MUFA (monounsaturated fatty acid): C14:1, C15:1, C16:1, C17:1, C18:1n-9, C20:1.

^5^PUFA (polyunsaturated fatty acid): C18:2n-6, C18:3n-6, C18:3n-3.

^6^HUFA (highly unsaturated fatty acid): C20:3n-6, C20:3n-3, C20:4n-6, C20:5n-3, C22:6n-3.

^7^n-3 FA (n-3 fatty acid): C18:3n-3, C20:3n-3, C20:5n-3, C22:6n-3.

^8^n-6 FA (n-6 fatty acid): C18:2n-6, C18:3n-6, C20:3n-6, C20:4n-6.

**Table 5:** Fatty acid composition (% total fatty acids) in muscle of juvenile *E. sinensis* fed different experimental diets.

Fatty acids	Experimental diets^1^								Two-way ANOVA (*p* value)^2^		
	FO	FO-PC	PO	PO-PC	SO	SO-PC	OO	OO-PC	PCL	LS	PCL × SL
C14:0	0.53 ± 0.03^b^	0.58 ± 0.05^b^	0.78 ± 0.06^c^	0.82 ± 0.07^c^	0.34 ± 0.02^A,a^	0.44 ± 0.01^B,a^	0.40 ± 0.02^B,a^	0.31 ± 0.02^A,a^	0.367	<0.001	0.125
C14:1	0.09 ± 0.01^b^	0.08 ± 0.02^a^	0.12 ± 0.02^c^	0.14 ± 0.03^b^	0.04 ± 0.00^a^	0.06 ± 0.01^a^	0.05 ± 0.01^a^	0.05 ± 0.01^a^	0.442	<0.001	0.629
C15:0	0.31 ± 0.01^B,b^	0.25 ± 0.02^A,d^	0.23 ± 0.03^b^	0.20 ± 0.01^c^	0.08 ± 0.04^a^	0.07 ± 0.00^a^	0.15 ± 0.01^a^	0.05 ± 0.00^b^	0.069	<0.001	0.446
C15:1	0.03 ± 0.00^a^	0.04 ± 0.01^a^	1.23 ± 0.58^b^	0.69 ± 0.36^b^	0.02 ± 0.00^A,a^	0.03 ± 0.00^B,a^	0.02 ± 0.00^a^	0.03 ± 0.00^a^	0.227	<0.001	0.276
C16:0	17.49 ± 0.15^B,c^	15.55 ± 0.62^A^	14.96 ± 0.54^ab^	14.85 ± 0.69	13.86 ± 0.41^A,a^	15.27 ± 0.23^B^	15.28 ± 0.41^b^	14.78 ± 0.35	0.381	0.001	0.007
C16:1	5.81 ± 0.55^b^	5.74 ± 0.58	4.88 ± 0.70^b^	4.36 ± 0.93	2.86 ± 0.17^A,a^	5.41 ± 0.35^B^	5.15 ± 0.29^b^	4.66 ± 0.23	0.345	0.041	0.024
C17:0	0.37 ± 0.01^A,c^	0.41 ± 0.01^B,b^	0.25 ± 0.03^a^	0.27 ± 0.04^a^	0.30 ± 0.01^b^	0.28 ± 0.02^a^	0.23 ± 0.01^a^	0.26 ± 0.01^a^	0.166	<0.001	0.377
C17:1	0.39 ± 0.01^c^	0.40 ± 0.01^b^	0.26 ± 0.00^a^	0.30 ± 0.03^a^	0.29 ± 0.01^ab^	0.31 ± 0.01^a^	0.32 ± 0.02^b^	0.31 ± 0.01^a^	0.225	<0.001	0.537
C18:0	7.18 ± 0.32^ab^	8.48 ± 0.60^b^	6.93 ± 0.43^a^	6.66 ± 0.68^a^	7.95 ± 0.27^b^	7.29 ± 0.24^ab^	6.51 ± 0.25^a^	7.22 ± 0.28^ab^	0.375	0.038	0.084
C18:1n-9	24.64 ± 0.39^b^	23.41 ± 0.84^a^	26.04 ± 1.06^b^	25.75 ± 1.13^a^	21.36 ± 0.51^A,a^	22.55 ± 0.38^B,a^	38.57 ± 0.53^c^	35.35 ± 1.09^b^	0.278	<0.001	0.020
C18:2n-6	13.53 ± 0.39^a^	14.33 ± 0.30^a^	15.78 ± 0.46^b^	16.03 ± 0.50^ab^	31.95 ± 0.40^d^	29.51 ± 1.03^c^	16.97 ± 0.28^c^	16.75 ± 0.48^b^	0.300	<0.001	0.022
C18:3n-6	0.07 ± 0.00^B^	0.03 ± 0.00^A,a^	0.08 ± 0.01	0.10 ± 0.01^b^	0.08 ± 0.00^B^	0.03 ± 0.01^A,a^	0.08 ± 0.00^B^	0.03 ± 0.00^A,a^	<0.001	<0.001	<0.001
C20:1	0.05 ± 0.00^B,b^	0.03 ± 0.01^A,b^	0.10 ± 0.01^c^	0.07 ± 0.01^c^	0.01 ± 0.00^B,a^	0.00 ± 0.00^A,a^	0.01 ± 0.00^B,a^	0.00 ± 0.00^A,a^	0.001	<0.001	0.226
C18:3n-3	1.63 ± 0.09^a^	1.55 ± 0.06^a^	16.68 ± 0.88^b^	14.80 ± 0.99^b^	1.80 ± 0.05^B,a^	1.52 ± 0.04^A,a^	1.91 ± 0.04^a^	1.90 ± 0.01^a^	0.119	<0.001	0.208
C22:0	0.23 ± 0.01^B,b^	0.18 ± 0.01^A,b^	0.21 ± 0.01^B,b^	0.06 ± 0.01^A,a^	0.03 ± 0.00^a^	0.04 ± 0.00^a^	0.04 ± 0.00^a^	0.04 ± 0.00^a^	<0.001	<0.001	<0.001
C20:3n-6	1.07 ± 0.04^A,a^	1.30 ± 0.04^B,b^	0.98 ± 0.06^a^	0.96 ± 0.04^a^	1.91 ± 0.04^B,b^	1.60 ± 0.05^A,c^	1.06 ± 0.07^a^	1.27 ± 0.10^b^	0.526	<0.001	<0.001
C20:3n-3	1.97 ± 0.10^a^	2.28 ± 0.08^a^	2.51 ± 0.03^A,b^	2.92 ± 0.07^B,bc^	3.13 ± 0.07^c^	2.73 ± 0.18^b^	2.64 ± 0.15^A,b^	3.21 ± 0.06^B,c^	0.007	<0.001	0.001
C20:4n-6	0.65 ± 0.04^b^	0.70 ± 0.06^c^	0.59 ± 0.05^B,b^	0.38 ± 0.02^A,b^	0.20 ± 0.12^a^	0.07 ± 0.02^a^	0.11 ± 0.01^B,a^	0.08 ± 0.00^A,a^	0.042	<0.001	0.089
C20:5n-3	13.01 ± 0.49^d^	13.99 ± 0.99^c^	5.77 ± 0.55^a^	6.84 ± 0.88^a^	10.16 ± 0.25^B,c^	8.06 ± 0.72^A,ab^	7.30 ± 0.26^A,b^	9.93 ± 0.47^B,b^	0.215	<0.001	0.025
C22:6n-3	10.90 ± 0.39^c^	10.59 ± 0.56^b^	2.56 ± 0.23^A,a^	3.31 ± 0.09^B,a^	4.24 ± 0.13^B,b^	3.33 ± 0.34^A,a^	3.00 ± 0.15^A,a^	3.98 ± 0.07^B,a^	0.658	<0.001	0.068
SFA^3^	26.08 ± 0.31^b^	25.44 ± 0.54^b^	22.79 ± 1.15^a^	22.52 ± 0.80^a^	22.56 ± 0.24^a^	23.52 ± 0.52^a^	22.74 ± 0.67^a^	22.76 ± 0.23^a^	0.966	<0.001	0.541
MUFA^4^	30.97 ± 0.95^b^	29.81 ± 1.75^a^	32.59 ± 1.28^b^	31.96 ± 1.65^a^	24.72 ± 0.87^A,a^	29.44 ± 0.53^B,a^	44.28 ± 0.74^B,c^	39.37 ± 0.72^A,b^	0.592	<0.001	0.012
PUFA^5^	15.23 ± 0.45^a^	15.94 ± 0.22^a^	32.54 ± 1.26^c^	30.92 ± 1.00^c^	33.83 ± 0.41^B,c^	31.05 ± 1.01^A,c^	18.96 ± 0.31^b^	18.67 ± 0.56^b^	0.050	<0.001	0.088
HUFA^6^	27.72 ± 0.98^c^	28.81 ± 1.77^b^	12.31 ± 0.83^a^	14.22 ± 1.43^a^	19.88 ± 0.44^B,b^	15.79 ± 1.28^A,a^	14.13 ± 0.64^A,a^	18.48 ± 0.66^B,a^	0.361	<0.001	0.016
n-3 FA^7^	27.63 ± 0.94^c^	28.44 ± 1.58^b^	27.19 ± 0.91^c^	27.51 ± 0.66^b^	19.58 ± 0.47^B,b^	15.64 ± 1.18^A,a^	14.88 ± 0.58^A,a^	19.11 ± 0.62^B,a^	0.625	<0.001	0.008
n-6 FA^8^	15.33 ± 0.44^a^	16.32 ± 0.38^a^	17.43 ± 0.44^b^	17.63 ± 0.70^a^	34.07 ± 0.36^B,c^	31.21 ± 0.95^A,b^	18.23 ± 0.33^b^	18.15 ± 0.54^a^	0.278	<0.001	0.008
n-6/n-3	0.56 ± 0.03^a^	0.58 ± 0.03^a^	0.65 ± 0.04^a^	0.64 ± 0.02^a^	1.74 ± 0.06^c^	2.09 ± 0.27^c^	1.24 ± 0.03^B,b^	1.00 ± 0.04^A,b^	0.613	<0.001	0.016

*Note:* Data are expressed as mean ± SEM (standard error of the mean) (*n* = 6). Values in the same line with different superscripts are significantly different (*p* < 0.05). ^AB^ Indicates a significant difference between phosphatidylcholine levels within the same lipid source (*p* < 0.05). ^abc^ Indicates a significant difference between lipid sources within the same phosphatidylcholine level (*p* < 0.05).

^1^FO, fish oil and without phosphatidylcholine; FO-PC, fish oil and 3% phosphatidylcholine; PO, perilla oil and without phosphatidylcholine; PO-PC, perilla oil and 3% phosphatidylcholine; SO, safflower oil and without phosphatidylcholine; SO-PC, safflower oil and 3% phosphatidylcholine; OO, olive oil and without phosphatidylcholine; OO-PC, olive oil and 3% phosphatidylcholine.

^2^PCL, phosphatidylcholine level; LS, lipid source; PCL × SL, phosphatidylcholine level × lipid source.

^3^SFA (saturated fatty acid): C14:0, C15:0, C16:0, C17:0, C18:0, C22:0.

^4^MUFA (monounsaturated fatty acid): C14:1, C15:1, C16:1, C17:1, C18:1n-9, C20:1.

^5^PUFA (polyunsaturated fatty acid): C18:2n-6, C18:3n-6, C18:3n-3.

^6^HUFA (highly unsaturated fatty acid): C20:3n-6, C20:3n-3, C20:4n-6, C20:5n-3, C22:6n-3.

^7^n-3 FA (n-3 fatty acid): C18:3n-3, C20:3n-3, C20:5n-3, C22:6n-3.

^8^n-6 FA (n-6 fatty acid): C18:2n-6, C18:3n-6, C20:3n-6, C20:4n-6.

## Data Availability

Data supporting this research article are available upon request.
